# Open Hardware: A Role to Play in Wireless Sensor Networks?

**DOI:** 10.3390/s150306818

**Published:** 2015-03-20

**Authors:** Roy Fisher, Lehlogonolo Ledwaba, Gerhard Hancke, Carel Kruger

**Affiliations:** 1 Advanced Sensor Networks Research Group, University of Pretoria, Lynwood Road, Pretoria 0083, South Africa; E-Mail: ckrugerl@csir.co.za; 2 Department of Computer Science, City University of Hong Kong, 83 Tat Chee Avenue, Kowloon Tong, Hong Kong; E-Mail: gp.hancke@cityu.edu.hk; 3 Meraka Institute, Council for Scientific and Industrial Research South Africa, Meiring Naude Road, Pretoria 0184, South Africa; E-Mail: lehlogonolo.ledwaba@yahoo.com

**Keywords:** Internet of Things, industrial networks, machine to machine, smart grid, wireless sensor network

## Abstract

The concept of the Internet of Things is rapidly becoming a reality, with many applications being deployed within industrial and consumer sectors. At the ‘thing’ level—devices and inter-device network communication—the core technical building blocks are generally the same as those found in wireless sensor network implementations. For the Internet of Things to continue growing, we need more plentiful resources for building intelligent devices and sensor networks. Unfortunately, current commercial devices, e.g., sensor nodes and network gateways, tend to be expensive and proprietary, which presents a barrier to entry and arguably slows down further development. There are, however, an increasing number of open embedded platforms available and also a wide selection of off-the-shelf components that can quickly and easily be built into device and network gateway solutions. The question is whether these solutions measure up to built-for-purpose devices. In the paper, we provide a comparison of existing built-for-purpose devices against open source devices. For comparison, we have also designed and rapidly prototyped a sensor node based on off-the-shelf components. We show that these devices compare favorably to built-for-purpose devices in terms of performance, power and cost. Using open platforms and off-the-shelf components would allow more developers to build intelligent devices and sensor networks, which could result in a better overall development ecosystem, lower barriers to entry and rapid growth in the number of IoT applications.

## 1. Introduction

The Internet of Things is a concept in which more devices (objects/things) will be communicating across the Internet than human users [1]. These connected and communicating devices will enable wide ranging applications. Already, applications from building automation [2] to smart-agriculture [3] have been deployed and tested, with much research being done into the next big thing within this new ecosystem [4–7].

Along with the increase in the interest and research devoted to the Internet of Things, a new development strategy has become more prominent in the Internet of Things ecosystem [8]. The development of both hardware and software using an ‘open-source’ approach has gained much attention [9]. Open source is the idea that all design and implementation documents get released in an open forum; it is, however, still possible for the original creator to control the licensing rights of the original design. Open source can extend to both hardware and software. In software, this includes all lines of code; in hardware, this typically includes documents describing the implementation. Open source hardware is a more recent development than open source software. Open source not only allows designers to create their own devices and systems from existing proven designs, but enables companies to sell complete open source systems to consumers. This allows for the consumer to design and improve the system as they see fit, but giving them a concrete standard and working starting point, typically known as a COTS (commercial off-the-shelf) system. One important aspect that needs to be considered is the use of open source technologies to implement the solutions required for the Internet of Things. Open source technologies and systems have a number of advantages and disadvantages [10]. One of the major advantages is that the system is generally designed with the wider community in mind; this means that compatibility issues are less likely. This model does lend itself to abuse, and all open source software and hardware should be thoroughly tested before being applied in highly sensitive applications.

Currently, the Internet of Things is an attractive idea for industrial applications [11,12]. Allowing data acquisition across multiple facets of an industrial life cycle could greatly increase efficiency, as well as allow the implementer a greater deal of insight and control over their industrial activities [13]. A possible application scenario would involve a great deal of embedded devices in a single environment. The major concerns for industrial implementations of the Internet of Things are: capability, reliability and cost [14]. With the increasing following and emerging standards for wireless communication, learning from and improving upon old technologies will allow for the creation of platforms that will still be developmentally relevant in the future. This will allow for developer communities to build and establish code bases and standards on which future platforms would be based. This would go a long way in being able to establish hardware and software standards for the Internet of Things.

However, the availability and cost of intelligent ‘things’ are arguably inhibitors of very large-scale adoption. More inexpensive technology could be used, such as radio frequency identification (RFID), but apart from identification and data capture, this does not truly realize an intelligent and connected device. Implementations of the Internet of Things designed for a specific application also appear to have seen little movement forward in terms of design updates. In this paper, we focus on the possibility that the development of supporting technologies for the Internet of Things could be based on generic and open source technologies that can be molded to suit the required task. It is our view that this approach would more rapidly increase the number of devices within the Internet of Things. Raspberry Pi is an example of such a device that could potentially be used in a number of applications [15,16]. However, a comparative study has not yet been completed as to the full capabilities of Raspberry Pi or similar platforms, when compared to ‘built-for-purpose’ Internet of Things devices, such as commercial sensor network nodes and gateways. The ease with which new technologies, such as Raspberry Pi, Beaglebone and the Arduino board, can be expanded, through modular expansion boards, and built upon will allow for an easy to implement and robust application using these devices. The application of modern, modular technologies will allow for an increase in the flexibility of the Internet of Things [17]. These technologies are aiming first and foremost to be generic, allowing for a more standardized approach to the Internet of Things. Designing generic technologies, which follow clearly predefined standards, will allow for a large degree of interoperability between all technologies. Industrial and consumer electronics have also reached a stage where the designer can easily and quickly use COTS modules and reference designs to build advanced embedded devices, which arguably provide comparable or improved performance in terms of power consumption, communication and processing resources when compared to built-for-purpose devices.

We believe that these advantages weigh heavily in the case for the application of open source technology to the Internet of Things. In a building automation example, the developer could deploy a relatively inexpensive open source COTS base system (Raspberry Pi) with a suitable modular communication capability (ZigBee) and easily design his own application to run on this system. As new communication technologies become available through the years, a simple upgrade to the communication module of the application is possible without completely redesigning the application or changing the already deployed base system [18]. This makes the application more flexible, cost effective and capable over the long term. Keeping these advantages in mind, we have decided to examine the possibility of taking open source Internet of Things technologies and applying them to wireless sensor networks [19].

Due to these advantages, one of the most appealing examples of the combination of the two separate paradigms is in the application of WSN and Internet of Things technology to the application of smart grids [20]. The focus is not on creating a hardened technology for important tasks within a factory environment. The idea is to introduce the Internet of Things technology gradually by initially using it in non-critical systems within a wireless sensor network. Using this design approach, we believe the open source concept is future proof, uses an advanced open source operating system, is inexpensive and as good as built-for-purpose components.

In order to provide some credibility for our belief, we tested built-for-purpose devices against both open source components and a COTS rapidly prototyped device that was created, in conjunction with the CSIR (Council for Scientific and Industrial Research in South Africa (http://www.csir.co.za/), using up to date components. We also created a testbed allowing us to benchmark our open source choices against the industry built-for-purpose devices that are currently available. The final aim of our paper is to provide an overview of all of the different technologies that are available and, through our testbed, give a blueprint to how these technologies can be used together. The paper details a cross-section of the most promising options that are available for Internet of Things applications. Additionally, these options are compared to some of the devices that were part of the first generation of sensor network technologies. This was done to act as a gauge of the progress that has been achieved through the development of the technologies related to the Internet of Things. We also created a device from COTS components, in order to provide a simple guide to better show the possibilities available when implementing and designing Internet of Things applications, especially when creating it for specific application areas.

## 2. Architecture

**Figure 1.**
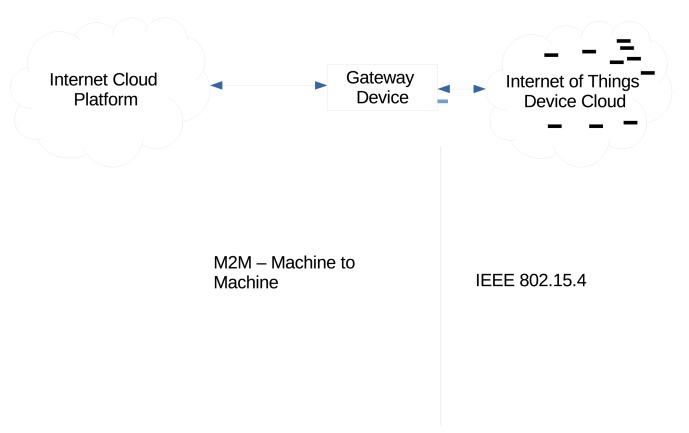
Internet of Things generic network implementation.

A generic implementation of the Internet of Things can be seen in Figure 1. The generic implementation shows a simple view of an embedded Internet of Things implementation. The Internet of Things device cloud is a traditional wireless sensor network. In this simple view, the gateway device would communicate with one of the many platform options that are available through standard technologies allowing for Internet connectivity [21].

In the image, two distinct networking zones can be seen. The division between these two networking zones exist because of the requirements of the devices that inhabit these areas of the architecture. One method of allowing these different networking zones to communicate is to make use of a gateway routing device.

### 2.1. Internet of Things Device Cloud

The device cloud is the implementation of a traditional wireless sensor network (WSN). These wireless sensors typically have two major functions: they allow for the sensing of the environment, and sometimes, they allow for the WSN to act on the environment (through an actuator).

The Internet of Things device cloud will consist of nodes that have at least the following capabilities:

- Self organizing, allowing for the determination of routes, as well as handling failed nodes;
- Protection of the data sent between the nodes and gateway devices;
- Communication capabilities for a large number of devices.

Some of the embedded protocols that these devices make use of are proprietary, and the gateway devices that will be used will need to be able to communicate across the chosen set of protocols.

### 2.2. Gateway

This section of the architecture for the Internet of Things typically involves the use of a gateway device to communicate and, in some cases, translate the device cloud communications onto the wider Internet, thereby allowing any Internet-capable device to communicate with the sensor and actuator device cloud [22,23]. Many options exist for the gateway devices. These gateway devices will have the capabilities of allowing for the translation between the Internet of Things device cloud and the cloud-based platform. These gateway devices will typically spend most of their time performing tasks similar to a routing device. The application of security features, such as encryption, to the data from the device cloud to the platform will also fall upon this gateway device. These gateway devices must be able to communicate with conventional network devices, as well as the low-power network devices embedded within the device cloud.

### 2.3. Cloud-Based Platforms

The back-end server devices are generally deployed in two separate ways: either the servers are deployed in-house or the developers use a platform to manage the server devices. These platforms are typically deployed on the cloud. This makes the deployment more elastic and, in some circumstances, more reliable. Some of the cloud platforms that are currently deployed are gaining more and more acceptance worldwide and becoming the standard method for creating Internet of Things applications. The two approaches both have their advantages and disadvantages. A number of articles have been written regarding this proposed structure [24]. A number of platform options exist, namely Axeda, Bugswarm, Carriots, Thingspeak and Xively. The purpose of this area is to collect, analyze and present the data in a method that the end user can both understand and make use of. This area is therefore primarily concerned with the presentation of the data to the client.

## 3. Nodes

Many different approaches have arisen to creating an Internet of Things application. Some of the current ranges of embedded enabling devices for the Internet of Things device cloud are introduced below [25].

We noticed that the divide between open source and closed source seems to be that the newer devices follow an open source approach, whereas the older versions follow a closed source approach. Waspmote, Lotus, Sprouts, Firefly and TelosB are open source. MICAz and Imote2 are closed source devices. A few of the devices mentioned as open source are not completely open source due to external problems and the inability to use the advantages of being an open source device. A good example of this is the TelosB device: the ability to advance or add attachments to the device is limited because of the design. Therefore, although being an open source device, due to external complications, it does not gain the advantages of other open source devices and is therefore very similar to a closed source device.

- Waspmote Pro
  Waspmote Pro is a new open source, wireless sensing node from cooking hacks by Libelium, improving on their initial Waspmote node [26]. Waspmote Pro can be used in various industries, such as gas and events monitoring, smart metering, agriculture and radiation detection, owing to a number of sensor boards available for use with the node. This node falls under our built-for-purpose devices, as it has been designed for a specific task and contains specific sensors and equipment.
- Lotus
  Lotus is an advanced WSN node built around the ARM Cortex M3, 32-bit processor [27]. It is fitted with a 802.15.4 compliant on-board radio associated with the ZigBee protocol. The Lotus is well suited for applications requiring acoustic, video, vibration and other high-speed sensor data, condition-based maintenance, industrial monitoring and analysis and seismic and vibration monitoring.
- Sprouts
  The Sprouts node is a developing node from Queens University [28]. Developed as part of industry-related research, the node has attracted attention in the oil and gas mining, steel production and power grid monitoring sectors as an event monitoring node. This is one of the nodes that falls under our open source options with many expansion capabilities.
- Firefly 2.2
  Developed at Carnegie Mellon University, the Firefly 2.2 is a real-time sensor networking node designed for use in industrial control and automation, smart home monitoring, inventory and personnel tracking and hazardous environment monitoring, as well as general embedded system education [29]. This is one of the nodes that falls under our open source options with many expansion capabilities.
- Older Options
  The newer options that have been discussed above are not the only options that are available. The other options to be discussed below are: TelosB, MICAz and Imote2. There are a number of other options that could be discussed, and a complete list of node options are available [25].
  Some other examples are WeC, Rene, Dot Mica, Spec, Cricket, EyesIFX, Tmote Sky, Shimmer, Stargate, Sun SPOT, IRIS, NetBridge, BTNode, V-Link and a few others [25]. Many of these examples are closed source. These devices were all created before 2009 and can be traced back to the designs used within TelosB, MICAz or Imote2. We have therefore decided to make use of these three devices as representative of the older devices that are available. The oldest versions are well represented by TelosB and MICAz (two of the first iterations of the Internet of Things), and through the years, the upgrades can all be represented through Imote2. These decisions were made after carefully studying the components used to create the different iterations of all of these available devices.
- Rapidly Prototyping New Devices
  Alternative options to the ones listed do exist. One of the major options that we have is to create our own device by making use of COTS components [30]. This allows us to personalize our devices to perfectly suit our requirements. The advantage gained from being able to create a node that specifically addresses your requirements must be done with the other devices in mind. The huge range of technologies used to create the Internet of Things often means that, within a single application, a large range of technologies are used. For example the 802.15.4 protocol is not the only protocol being implemented within this section of the architecture. Industrial production lines using the more mature WiFi protocol also exist [31]. WiFi technology is based on the 802.11 protocol and has been in use in both residential and industrial applications for many years. The 802.11 protocol, although capable of being implemented within the device cloud section of the Internet of Things, was originally designed for implementation within devices with high resource allowance (power, throughput and large memory space) [32]. The standard has evolved, and the current implementation, 802.1 lac, focuses on high data throughput and multiple access [33]; both are less important for the Internet of Things device cloud, where a large number of devices communicate irregularly. Using a device such as Raspberry Pi and a plugin XBEE or WirelessHart device will enable a powerful range of capabilities for an embedded sensor. These are a few of the considerations that must be taken into account when developing your own node.

**Figure 2.**
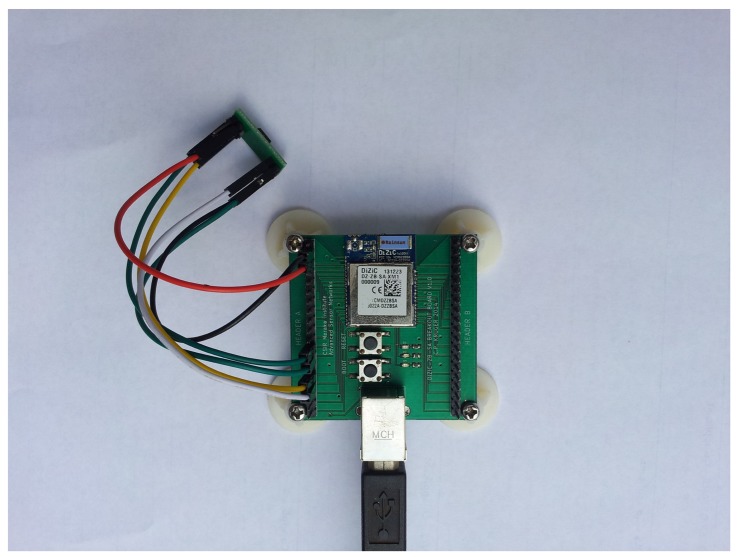
The rapidly prototyped Council for Scientific and Industrial Research (CSIR) Internet of Things node.

**Figure 3.**
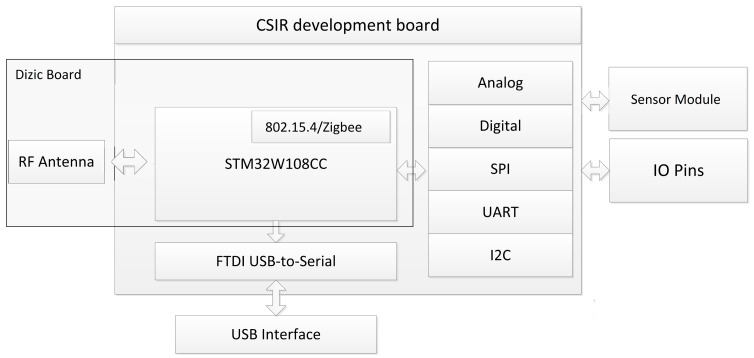
The CSIR Internet of Things node functional diagram.

Creation of your own node requires only a basic understanding of electronics, communication interfaces and embedded programming. To illustrate the feasibility of rapidly prototyping a self-built solution, we created a simple node to test alongside the nodes discussed above. The node that we prototyped is based on a DiZiC module (http://dizic.com/), with our own simple ‘break-out’ board and additional communication and power circuitry. The node has full communication capabilities based on the IEEE 802.15.4/ZigBee protocol stacks. The developed node can be seen in Figure 2, and the functional diagram is given in Figure 3. This shows the simple design of the implemented node and the range of communication options available to the node. The main chip was chosen to ensure a wide range of compatibility with as many of the available Internet of Things operating systems as possible. The other components were chosen to ensure that the device remained as cost effective as possible, but could still perform the required operations. An advantage of making use of such a design is to ensure that the device meets one's individual requirements and is able to meet the goals of one's application, but remain easy to both replace and upgrade. This ensures that the entire application remains robust, inexpensive and flexible, all of which ensure a future-proof design. This node that we have created is capable of performing all of the functions that the other devices can achieve and when compared against a number of the available options and at a much lower cost, even when considering that we were ordering small quantities.

### 3.1. Device Comparison

The primary comparison of the devices will look at their basic functionality and capability [28]. The initial testing will look at the power consumption of each of the devices.

The list of devices above can be broken down into two separate categories: traditional wireless sensor network nodes and non-traditional (devices that were not originally created to perform the role of a wireless sensor network node). Raspberry Pi and Beaglebone are the non-traditional WSN nodes, as they have not been implemented with the limitations of the WSN in mind. These devices have not been developed to use the minimal amount of power, *etc.* This means that these devices perform very well in certain aspects of our benchmarking.

#### 3.1.1. Power Comparison

**Figure 4.**
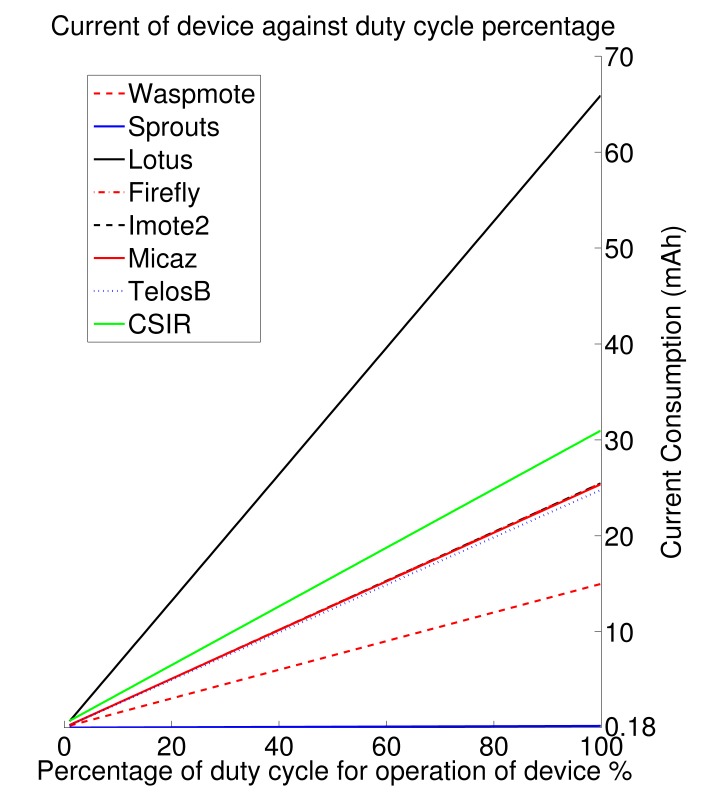
Wireless sensor nodes' current consumption against duty cycle.

**Figure 5.**
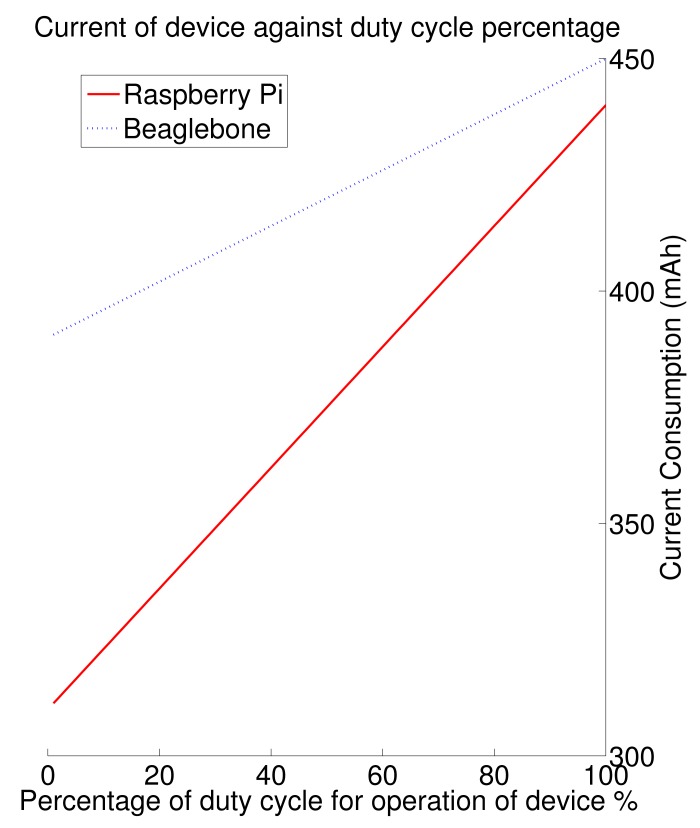
Non-traditional nodes' current consumption against duty cycle.

The two graphs seen in Figures 4 and 5 show the power consumption of all of the tested devices. These graphs show the current consumption (in mAh) against the duty cycle (% of time spent sending and receiving information) for these individual Internet of Things nodes. For the time that the nodes are not operational, the device spends its time in sleep mode, attempting to conserve as much energy as possible. The most economical of all of the devices is the Sprouts device. This device successfully achieves less than 180 μAh in full active mode (continuously awake). Some of the other economical devices are Waspmote, which achieves a maximum of 15 mAh in full active mode. The older devices that have been compared here all perform very similarly. These are TelosB and the MICAz; they both achieve around 25 mAh for full awake operation. The Firefly node achieves a similar current requirement of 24.8 mAh for continuous operation. These devices are followed closely by Imote2 and Lotus platform, each achieving 44 mAh and 66 mAh, respectively, for continuous operation. Using these graphs and by knowing the power limitations of the application being designed for, a suitable node can be chosen beforehand. This allows for adequate planning and can help prepare a suitable budget when deciding on which node to use for the Internet of Things application.

As can be seen from the graphs shown above, our rapidly prototyped COTS node has only slightly higher power requirements, mostly due to the more powerful processor that is included on the board, than a cluster of current sensor node options. The node achieves 31 mAh when operating at 100% and 400 nAh when in sleep mode. The sleep mode current is high for the device, but the operational current is similar to what is required for the other nodes.

The power consumption of the non-standard nodes (by non-standard, we mean any device that is not originally created for use within the Internet of Things/WSNs) is significantly higher. The sleep and non-sleep of these nodes is defined differently from the sleep and non-sleep of the traditional nodes. A traditional WSN node sleep typically involves a state in which most power consuming hardware is off and waiting for a wake command, either from the onboard processor or another device in the network. The sleep for Raspberry Pi and Beaglebone is defined as a state of rest, where little to no processing is occurring (an expansion board for Raspberry Pi can be added, which will enable a WSN node, like sleep mode). The traditional sleep (for WSN without the expansion board) can be achieved, but it is very difficult to wake the device, and it takes a significant length of time longer than the more traditional nodes. As can be seen from Figure 5, Raspberry Pi consumes 44 mAh for continuous operation, and Beaglebone consumes 450 mAh for continuous operation.

#### 3.1.2. Capabilities Comparison

In the current world of computing, more cost typically gives you greater capability. A comparison was completed between the cost of the nodes and the processing capability that is received for the cost. Processing capability is not the only performance-based metric that can be used. This metric was chosen, as it is generally the one most linked to the cost of the node and potentially the one with the most impact on actual performance.

**Figure 6.**
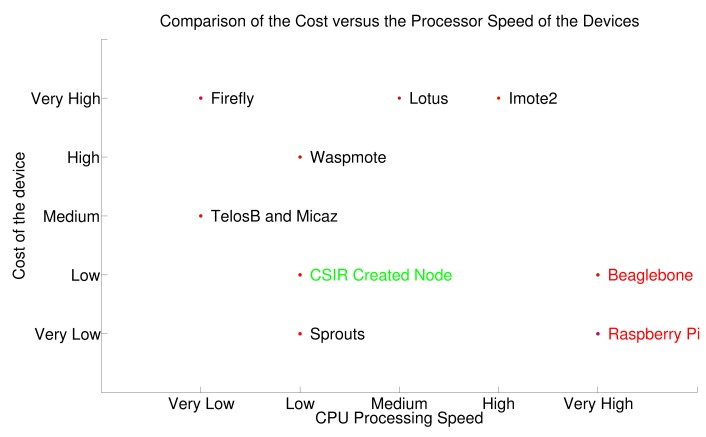
Comparison of processing speed against cost for WSN nodes (commercial off-the-shelf (COTS) and open platforms indicated in green and red, respectively).

The cost of the Firefly device is currently not known, as it is still under development. The Sprouts device is open source, and although the cost of the completed device is not available, the bill of materials cost was used, as a capable individual could in theory create one of these devices themselves. Surprisingly, in this comparison (Figure 6) the most powerful devices are also the least expensive. The Sprouts node is the least expensive complete WSN node, with our own rapid prototype device coming in as being one of the least expensive available, as well. Although both Sprouts and the CSIR-made device are low on processing and power, depending on the application, this should not be a drawback from the implementation within a wireless sensor network. The low power requirement will allow for long-term deployments within Internet of Things applications, such as those within embedded environment monitoring applications, and with a careful design, the low processing power will not negatively affect the performance of the application.

The next two least expensive are Raspberry Pi and Beaglebone. Both of these devices will require a XBee module (or similar) for wireless communication. These modules cost about $30–$40. This will not change the ranking of these two devices on the scale. This graph shows that some of the least powerful devices are in fact the most expensive. The Lotus node and the Imote2 node both cost $300, a massive sum to be paying for a WSN node. The built-for-purpose devices end up being expensive, possibly due to the ease of plug and play capability. The additional cost for Raspberry Pi and Beaglebone is getting the devices to communicate successfully with the network through the general purpose input output (GPIO) serial ports. Due to the open source nature, many tutorials and online examples exist to assist with this problem.

#### 3.1.3. Features Comparison

This comparison looks specifically at the hardware and software capabilities of the individual devices (costs shown in the table are rough guides, as large discounts can be achieved when ordering large quantities).

As can be seen, the features of the devices differ greatly. One important feature is the operating system of the node chosen. The operating system has an impact on the stability, capability and security of the device. A well-developed and continuously-updated operating system can allow for new powerful features to be deployed to the nodes without making any hardware changes. Raspberry Pi and Beaglebone both have many options available, and many of these options are well supported in terms of continuous updates. TinyOS and Contiki are both open source, with active communities driving development. NanoRK is an operating system created by Carnegie Melon University for use on their WSN node. Mote Runner is a set of tools to aid the running of the node from IBM [34]. Table 1 shows that the features of the devices can differ greatly. However, it also shows that the current communication choice for Internet of Things embedded nodes is the ZigBee protocol. This is due to the large number of devices that support this communication standard, as can be seen from Table 1. A number of the devices also include built-in onboard sensors that can be used for sensing applications, giving a reduction of the cost for certain applications when these onboard sensors are used. Some of the devices also include a range of GPIO ports available on the devices. These devices allow for advanced features to be added to the application. As can be seen, a range of features is available to the nodes.

It can be seen from the table that our node, although being a rapid implementation, has similar capabilities to the other nodes tested. This shows that the use of the circuits available and a simple working knowledge of general electronic skills can allow one to create powerful and inexpensive devices. Our node has full 32-bit capability, is coded in a standard language (C++) and has a range of expansion capabilities through the generic I/O pins. The node is also significantly less expensive than many of the built-for-purpose devices.

Table 1 shows as many of the capabilities of each of the individual devices. Some of the additional expansion capabilities are included in footnotes at the bottom of the page, and these contain links to developer web pages, showing a large list of expansion capabilities available for Raspberry Pi and Beaglebone.

## 4. Gateway

When considering the performance capabilities of gateway devices in an industrial implementation, the most important characteristic is communication performance. To prevent data bottlenecks and slow transmission when measuring performance, three key areas must be considered: reliability, throughput and response time [12].

Reliability measures the confidence in a transmitted packet being received by the cloud platform. For a gateway in an industrial application, high reliability is required. This enables the application implementer to be sure that transmitted data are received by the cloud platform. High reliability will ensure that data transfer rates are not negatively affected by retransmission of data across a low resource network. Typically, networks based on the 802.15.4 protocol stack do not implement a transmission control protocol [35]. This means that reliable transmission is not guaranteed by the communication protocol. A high reliability will allow for a greater degree of confidence that transmitted data have reached the destination. Reliability must also take into account the order in which the packets are received. In a time-sensitive implementation, which has limited resources, any additional processing must be avoided [36].

Throughput is a measure of the amount of data that the system can process per second. The gateway implementation will need to take data from one communication technology and retransmit on the other end. This will typically involve receiving data from a 6LoWPAN network and re-transmitting it on a normal IPv6 or IPv4 network. The higher the throughput of the device, the better for the implementation. Another measure of the throughput is the latency of the device. Latency can also be called response time. This is a measure of the time it takes for another device to respond. A low latency is preferred, as this means that the gateway is operating more efficiently.

Due to the nature of the information that will be transferred across the Internet by Internet of Things applications, the additional process of adding security to the communication needs to be benchmarked [37]. The three major requirements for the Internet of Things security is confidentiality, integrity and authentication [38,39]. These three requirements are known as the primitive security objectives. The processing requirements of these primitive objectives are extensive and will be a good benchmarking tool for the devices, but these devices will also be required to implement these objectives in communication in order to secure the communication [40]. Confidentiality is the ability to hide the data that are transmitted. Integrity is the ability to ensure that the data that are transmitted are the same as the data that are received. Authentication is used to ensure that the individuals communicating are the people that are expected to be part of the communication. Using all three of these objectives allows us to ensure data security when communicating.

To complete the benchmarking of the possible devices, two approaches could be used: built-in toolkits for benchmarking; or the creation of scripts that perform the required benchmarking.

### 4.1. Open Source Implementation

Raspberry Pi has often been labeled as a ‘hobby’ device [15]; this leaves questions as to whether or not this device is capable of operating as a gateway or in any time-/data-sensitive application. Due to the increasing community interest in Raspberry Pi, the device has undergone a large amount of testing and benchmarking already. Another device to consider for implementation is Beaglebone. This device has been benchmarked, but is not considered as an easy alternative to Raspberry Pi. The focus of the paper is to supply a current state-of-the-art for the implementation of an Internet of Things application. Beaglebone, although being a very capable device, has not seen the interest and development from third party individuals that Raspberry Pi has enjoyed. Beaglebone has a less well-supported operating system, known as Angstrom Linux. Although the operating system on both devices can be changed, the paper looks at the default design of the devices, and Raspberry Pi's Raspbian operating system has more support and software packages due to its Debian heritage.

During the benchmarking of the devices, it was shown that Beaglebone and Raspberry Pi perform on similar levels. Beaglebone lacks some of the easy expansion capabilities of Raspberry Pi. The best example is the ability of Raspberry Pi through a $40 board to be able to use Arduino shields. In order to easily use a Xbee module, a $6 board is required [41]. It was therefore decided that Raspberry Pi would represent the open source implementation of a gateway device.

### 4.2. Proprietary Implementation

The proprietary implementation of the gateway devices involves the use of the Digi Connectport devices acting as the gateway for the embedded Internet of Things sensors to the wider Internet. As discussed previously, the two devices that are tested are the Connectport X2 and the Connectport X4. Both of these devices have LAN and ZigBee networking capabilities. Unlike Raspberry Pi, these devices have a very limited set of customization options available. They both run the Digi-implemented operating software that has a specific focus on operating requirements [42,43].

### 4.3. Experimental Details

A set of experiments were conducted that showed the capabilities of Raspberry Pi with regards to the requirements for an open source gateway device, namely reliability, throughput and security. The aim of these experiments was to determine whether or not Raspberry Pi is a device capable of acting as a gateway for the Internet of Things in an industrial application. Tests were done to show each of the requirements and to measure Raspberry Pi's capabilities with regards to these requirements. Each of the tests will be discussed in the sections below. Due to the interest in Raspberry Pi, many benchmarking tests have already been completed. These benchmarking tests (specifically the OpenSSL tests) use the built-in benchmarking capabilities provided within the OpenSSL development kit. Although being a good performance comparison between the different devices available, it does not provide a real-world comparison of the devices. The benchmarking tools allow for uniform data to be worked upon in a structured and controlled loop only utilizing high-speed sections of onboard hardware (RAM). This is the ideal operating environment for benchmarking, but does not provide a real-world view of operational capabilities. A real-world implementation involves more operational calls to external input output devices and interrupts from other devices. This is why when completing the implementation, these external features were simulated within the code, allowing for a more realistic view of the real-world operational capabilities of the device.

#### 4.3.1. Reliability Test

The first set of tests were done to confirm the reliability of Raspberry Pi. Reliability testing was done using a widely-known network benchmarking tool. This is used to ensure that the packets sent and received are reliably delivered. It should be noted that our definition of reliability is somewhat limited. We have decided to only focus on the successful delivery of packets between the receiver and transmitter. This was to provide us with a solid starting point for comparison against the two security protocols tested later in the section. Although being a limited approach, it provides us with a good basis for testing the security protocols. The reliability could be affected by nodes moving in or out of the network range, as well as other possible faults that could arise. The test consisted of a laptop connecting to Raspberry Pi via the laptops WiFi and a USB WiFi receiver on Raspberry Pi. Raspberry Pi hosted a WiFi access point and was also connected to a desktop computer via the onboard Ethernet capabilities. Each of these separate networks was setup in a subnet. Raspberry Pi had IP forwarding enabled, switching the packets between the Ethernet and WiFi networks. Making use of the Iperf benchmarking suite, the performance was measured [44]. When measuring the reliability of the wireless communication, the distance of the transmitter from the receiver can play an important role. The tests were run at three distance intervals, with the laptop moving away from Raspberry Pi.

The Connectport devices are designed for reliability, as they are created to be deployed in an industrial environment. The Connectport devices have a similar setup structured for the reliability testing. Due to the wired nature of their communication, distance testing was not required.

#### 4.3.2. Throughput Tests

The throughput testing was completed using two separate tests. The raw data bandwidth was measured using the Iperf benchmarking suite for data of different sizes. Additionally, the Iperf benchmarking suite has the capability of simulating the communication of up to one hundred devices. A test was run that simulated the communication of a large number of devices across the WiFi channel to Raspberry Pi and onto the server connected via Ethernet. This simulation was run three times, and the average results were calculated. The latency of the communication was also measured by pinging the laptop and desktop computer through Raspberry Pi.

##### Secure Throughput Tests: Raspberry Pi Command Line

A Python script was created that could call the required OpenSSL command line tool. The Python script built the required command and then called the command using the built-in set of OpenSSL command line functions on Raspberry Pi. The command was executed on a file that was greater than 500 MB, and the time to complete the required security objective was measured. The authentication of the system is not included in the results below, as the times were consistently less than one second. This leads to unreliable results, so it is assumed that a key is used that is encrypted with the message as authentication. These tests were run a number of times, and the average throughput calculated. The tests were completed using a block size of 8,196 bytes. These tests assumed that the entire file has the required security objective applied and is then transmitted as an entire package by some underlying protocol, for example a TCP-based unencrypted communication. The underlying protocol will then break the file into packets as required. Once received, the entire file could be decrypted using a pre-shared key. For the integrity tests, the secure communication of the signature of the file was assumed to exist. The raw processing and throughput speeds are more important in terms of benchmarking Raspberry Pi. A simple solution is to transmit this information as part of the file in the first 16 bytes of the message. By doing this, no major additional processing is required; it will simply be the attachment of x (the size of the integrity proof) bytes onto the file. The aim of these tests is to determine the processing and communication time required to protect the communicated data for each of the primitive security requirements.

##### Secure Throughput Tests: Raspberry Pi Python Wrapper

This implementation broke up the file into equally-sized packets (1,024 bytes). Each packet then had a combination of integrity, confidentiality or authentication applied to it and was then transmitted. These tests were done to better simulate the size of data that would be received from the in-house network, as this is generally small packets from a large number of devices. The aim was to show the added overheard when dealing with the small-sized data packets that are typically going to be received and transmitted by Raspberry Pi. The M2Crypto Python wrapper was used to interface with the OpenSSL C functions [45]. An additional advantage of using a trusted and established wrapper for secure communication allowed us to confirm our results.

The next set of tests was completed using the best performing cipher suite and message digest to provide confidentiality, integrity and authentication. A system was then implemented that hashed, signed and finally encrypted each 1,024-byte message and transmitted it along the network. The message digest used was MD5, as this has proven to be a fast hashing algorithm. The signing algorithm was RSA-based, and the encryption was implemented using the AES 256 CBCalgorithm.

##### Secure Throughput Tests: Proprietary Implementation

Similar testing was completed for the the Digi devices. The testing allowed for secure sockets layer-based communication of data (built from the data collected from the embedded network) between the Digi device and a suitable standalone computer.

### 4.4. Results

#### 4.4.1. Reliability Tests

The results for the test can be viewed in Table 2. As can be seen from these results, Raspberry Pi is capable of achieving reliable data transmission at distances up to 10 m with very few packets either dropped or arriving out of order.

The proprietary Digi Connectport X2 and Digi Connectport X4 devices have reliability and response capabilities expected of a networking device in an implementation. The devices achieve a 0% packet drop rate and a very low response time, averaging <10 ms.

#### 4.4.2. Throughput Results

The results for the raw (unsecure) data throughput tests can be seen in Table 3. These data show that the larger the information being sent through Raspberry Pi, the more throughput is being achieved; the increase is due to the larger data sizes being able to stream at maximum speed for longer periods due to all of the communication, requiring setup time. The data also shows that Raspberry Pi does not have a large effect on the data flow, as the communication is limited by the WiFi standard used, namely 802.11g, which achieves a maximum of a 22-Mbps throughput [46].

For the Iperf communication of multiple device simulation, Raspberry Pi was capable of achieving an average throughput for 100 communicating devices of 15.8 Mbps.

The ping testing was repeated 10 times, and the average time to receive a response was 80.011 ms. The minimum response time was 12.669 ms, and the maximum response time was 205.143 ms. This large swing in response time could potentially negatively affect the performance of communication through Raspberry Pi. The large swing could be due to the single core processor that is included with Raspberry Pi. The response to one of the pings is delayed, because the processor is performing some other set of tasks (other processor interrupts) before it can return to the task of responding to the request.

##### Secure Throughput Results: Raspberry Pi Command Line

The results for the implementation applying only confidentiality are shown in Table 4. The data throughput using the recommended encryption cipher AES with a 256-bit key achieves 3.20268 MB/s. The two fastest performing algorithms are Blowfish and AES, both achieving greater than a 3-MB/s throughput. Tests were also completed with regards to the cipher chaining method. The best performing chaining method was ECB (electronic code book); this was expected, because of the simplicity. The next best performing chaining method was CBC. AES 256 using CBC achieved a throughput of 3.279 MB/s, whereas the ECB achieved a throughput of 3.421976 MB/s.

One important consideration is RC4; it would appear that by adding I/O operations, the throughput of this algorithm is greatly reduced. The operation of RC4 is delayed due to the I/O operations that need to be performed. Whereas without the file I/O, the RC4 stream cipher could rapidly process the data due to its stream nature, complementing the continuous loop cycle of the benchmark test. It is now being hampered by the low speed of the I/O operations, but the cipher is still able to perform at the fastest rate.

The results in Table 5 show the results achieved when adding integrity to the communication with the OpenSSL command line tool. As can be seen, the results for just integrity far outperform the throughput of adding confidentiality. The best performing hashing function was MD4, achieving a throughput of 28.82474 MB/s. These results are achieved by implementing the hash on the complete file and then transmitting the hash value along with the file.

**Figure 7.**
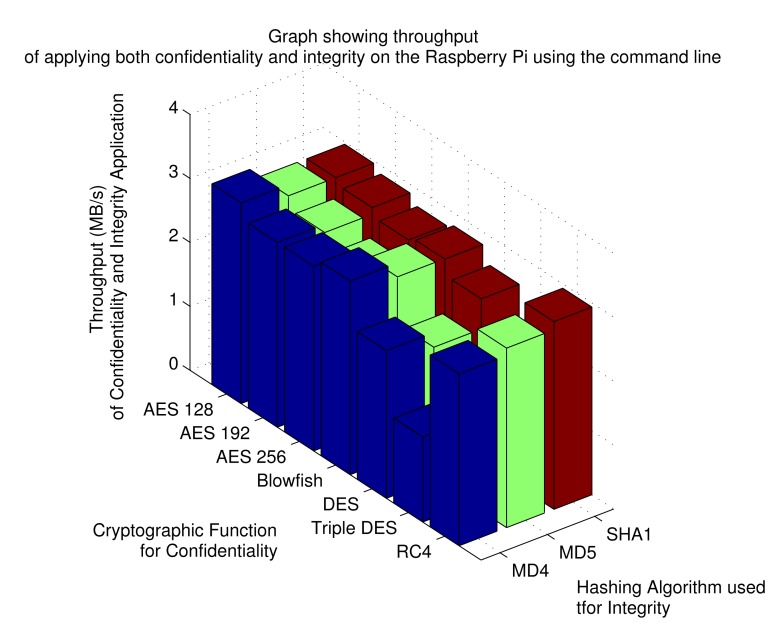
Throughput results when applying both confidentiality and integrity for command line application.

Figure 7 shows the results for the implementation of both integrity and confidentiality on the data sent from Raspberry Pi. These results show that a data throughput rate of 2.962641 MB/s is achievable using the AES 128 encryption with MD5. Similar speeds can be achieved using AES 128 with MD4 and RC4 with SHA1. The more secure implementation of AES 256 with MD4 achieves 2.852727 MB/s. These values show that by adding I/O operations, the speed of the system is slowed drastically. It is important to note that in these tests, authentication of the communication is handled by the knowledge of the pre-shared key.

##### Secure Throughput Results: Raspberry Pi Python Wrapper

The first set of results were for the implementation of the described system passing messages of a size of 1024 bytes, encrypting, then sending the data and can be found in Table 6. As can be seen, the throughput achieved is very similar to the throughput achieved for the previous implementation using the command line. Only CBC mode was tested, because from the previous experiments, it became clear that this was the best performing mode. It is clear from these results that AES and Blowfish again achieve the fastest throughput. This is expected and confirms the results found regarding the fastest cipher [47].

The results of applying all of the primitive security objectives using the results from the previous tests are that the throughput capability of applying all of the primitive security objectives was 1.5778 MB/s.

Tests were not implemented to confirm the capabilities of signing and hashing each protocol, as there is no point in applying each primitive security objective separately in a comparison test. One more test was completed. This test measured the throughput of Raspberry Pi when implementing confidentiality and integrity on the device. This test was done to mimic the operation using a static key for authentication. This test achieved a throughput of 1.764865 MB/s. This shows that the process of signing the information does not account for a very large percentage of the processing power of the processor. This confirms the results that where achieved in the first benchmarking results.

##### Secure Throughput Results: Proprietary Implementation

The results show that the X4 device performs significantly worse than the X2 device, achieving only 113 KB/s of data throughput compared to the X2 achieving 1.4 MB/s. Both of these devices perform worse than Raspberry Pi. This is surprising, since the two Digi devices are specifically designed for the application.

#### 4.4.3. Summary

Table 7 shows a summary of the throughput results achieved in the paper.

Raspberry Pi is capable of achieving a maximum of 2.962 MB/s when applying confidentiality and integrity to the data that are transmitted by using a Python script and the OpenSSL command line. This is equivalent to 23.696 Mb/s. If a small amount is subtracted for line inconsistencies and overhead from Internet Protocols used in Internet communication, Raspberry Pi is capable of interacting with a 20-Mb/s communication line.

The Python library implementation achieves a 1.5778 MB/s or 12.6224 Mb/s on Raspberry Pi. The additional inefficiency of interacting through the library could be the reason for this decrease in raw speed. However, this still outperforms the Digi devices when implemented using the correct algorithms.

The proprietary implementation achieves a maximum of 1.4 MB/s or 11.2 Mb/s when applied on the newer X2 device and 113 KB/s or 0.882 Mb/s when applied on the older X4 device.

This shows that Raspberry Pi is capable of being used as a gateway device for the Internet of Things. Raspberry Pi successfully outperforms the Digi devices for throughput. This shows that Raspberry Pi is capable of being implemented as a gateway device in an industrial application.

Although Raspberry Pi performs very well, the simplicity of a proprietary implementation could be very appealing to a system developer.

It should also be noted that applying security to Raspberry Pi does not hugely impact the throughput capability of the device, as can be seen from the small difference in throughput with or without secure communication. Raspberry Pi is capable of acting as a gateway device, due to its stable reliability, availability and its high throughput capability Raspberry Pi, even though it has a low resource processor, is able to successfully handle all of the tasks associated with the secure communication of information between multiple embedded devices and the wider Internet platform.

Our results show that an open source, inexpensive and simple solution is available for a gateway device based within the Internet of Things.

## 5. Platforms

Two primary examples of Internet of Things applications exist. The first option is to deploy the application using a centralized cloud-based server. The second option is to deploy a localized server within the organization and thereby have more control over the flow of their data [48]. Many of these systems already have industry partners and have begun to be deployed within some of the largest commercial institutions in the world. Using these platforms in this manner allows the application developer to focus his efforts on his application and instead leverage the power that these cloud-based systems allow for large amounts of data collection.

Typically, information is transmitted from the device to the gateway, and these data are then forwarded, with the required information (device ID, *etc.*), to the cloud platform, which collects and stores the data for access. This allows advanced data management features and analysis to be implemented easily and simply by the user and developer of the application.

The data that traverse the Internet of Things are typically going to be of extremely high importance [49]. Using the above example of a centralized server facilitating the collection of the information generated in a smart grid scenario, it can be seen that just on the basis of privacy, this collected information must be stored and transmitted using the current security standards. Examining the work in the gateway section and previously covered work, the secure transmission of the data has been covered [48].

The final stage is the storage of this information on the server side for later use. One of the most efficient ways to protect the information is to use local server-side encryption. By encrypting the data, only a user with the registered and required key can decrypt and, therefore, access the data [50]. This method allows for the protection of the information stored on the server and does not open up our system to a set of possible security flaws. Another method is to manage access to the data. By controlling who has access rights and enforcing a deletion policy if these access rights are traversed, our data can be securely protected [51].

Generally a built-for-purpose implementation will contain an in-house server that the local IT department has full control over. However, this method of deploying the platform for the application is both expensive and requires the purchasing of additional equipment.

A newer approach is to use the open source platforms that enable one's application to be built utilizing their platform as a service (PAAS). This allows the application implementers to focus rather on their own application and allows the platform to control the processing, network and storage requirements. This is generally done for a small fee or a limited free account does exist [48].

## 6. Conclusions

The Internet of Things is becoming a popular term in both the media and in the business world. The ability of these open source devices has been covered within the paper. This paper has shown that the devices could be used in place of a proprietary implementation. In some instances, the open source ‘cheaper’ devices perform better than the ‘expensive’ proprietary devices. This is encouraging as the inexpensive and open source devices could be a possible solution for the implementations of the Internet of Things.

One of the important devices that are benchmarked within the paper is Raspberry Pi. This device is inexpensive and easily upgradeable through simple expansion boards, allowing for varied and diverse implementations. Many of these advanced expansion boards and capabilities are due to the extensive support that Raspberry Pi has received from the development community. This has created a source of knowledge and useable program packages that have pushed Raspberry Pi into a truly multi-function device.

The ideal implementation from the results found above would be to use the Sprouts platform for the embedded device cloud and Raspberry Pi as the gateway device. By doing this, the advanced capabilities of Raspberry Pi are available, and the power saving of the Sprouts platform is utilized. This entire implementation is cost effective, as Raspberry Pi is $80 with full Xbee (ZigBee protocol capable module) capability, and each of the Sprouts platform devices will cost $20. This is much more inexpensive than any proprietary implementation that is available.

This set of results shows that an open source solution is a possibility and, in many cases, is often preferable to a built-for-purpose implementation. Using Raspberry Pi as a gateway device and either building one's own node or using the Sprouts node design, with some basic electronic skills, a powerful and cost-effective Internet of Things implementation is possible. We believe that this is the possible approach that could guide the future creation of Internet of Things devices.

The focus for the future is for the individual devices embedded within the device cloud to be directly connected to the Internet using the new Internet Protocol Version 6 addressing scheme. This is the focus for the future and is being carried out by research into areas, such as 6LoWPAN and COAP. 6LoWPAN is the protocol for converting the IPv6 protocol for use on low power personal area networks typically based on the 802.15.4 protocol. This allows for the embedded sensor or actuator to communicate directly to the cloud-based platform. In the future, this may be the preferred implementation setup.

## Figures and Tables

**Table 1.** Table showing features of the WSN nodes. GPIO, general purpose input output.

**Node**	**OS**	**CPU Architecture (bits)**	**CPU Speed (MHz)**	**Network Standard**	**No. of Sensors**	**Language**	**I/O Pins**	**Release Date**	**Cost ($)**
Waspmote	None	8	14.75	Various (Depends on Implementation)	2	C/C++	21	2013	210
Sprouts	Linux	32/8	32	ZigBee, BLE	2	c	0	2011	20
Lotus	Mote Runner/ TinyOS	32	10–100	ZigBee	0	nesC	0	2011	300
Firefly	NanoRk	8	7.3	ZigBee	0	C	20	2010	Un-known
Imote2	TinyOS	32	13–416	ZigBee	0	nesC	12	2006	300
MICAz	TinyOS	8	16	ZigBee	0	nesC	4	2004	100
TelosB	TinyOS or Contiki	16	16	ZigBee	3	nesC	16	2005	120
CSIR Node	Contiki	32	24	802.15.4/ZigBee	0	C/C++	17	2012	80
Raspberry Pi	Raspbian, Android, Many more	32	700	None, but expandable ^1^	0	Any for Linux OS	0 GPIO and UART, *I*^2^C SPI	2012	35
Beagle-bone	Angstrom, Raspbian, Many more	32	720	None, but expandable ^2^	0	Any for Linux OS	65 GPIO and UART, *I*^2^C SPI	2012	89

1 Raspberry Pi expansion board (http://elinux.org/RPi_Expansion_Boards);

2 Beaglebone expansion boards (http://elinux.org/BeagleBone_Community).

**Table 2.** Table summarizing the results from the reliability test with a Raspberry Pi.

**Distance**	**Result**
<1 m	0% packets dropped
5 m	0% packets dropped and 1 arrived out of order
>10m	0% packets dropped

**Table 3.** Table summarizing results from the throughput test with Raspberry Pi.

**Data Size**	**Result**
15 MB (megabytes)	11.5 Mbps (Megabits per second)
18.2 MB	15.2 Mbps
60 MB	21.5 Mbps
200 MB	22.2 Mbps

**Table 4.** Table summarizing results from Python and the command line implementation of communication with confidentiality.

**Confidentiality Algorithm**	**Throughput (MB/s)**
AES128 bit key	3.34744
AES 192 bit key	3.318837
AES 256 bit key	3.20268
Blowfish	3.317878
DES	2.590775
Triple DES	1.458663
RC4	3.58629

**Table 5.** Table summarizing results from Python and the command line implementation of communication with integrity.

**Integrity Algorithm**	**Throughput (MB/s)**
MD4	28.82474
MD5	24.85157
ripemd 160	15.347
SHA	19.09429
SHA1	21.68956
SHA 224	15.2319
SHA 512	8.411452
Whirlpool	1.566124

**Table 6.** Table summarizing results from the Python OpenSSL wrapper implementation of communication with confidentiality.

**Confidentiality Algorithm**	**Throughput (MB/s)**
AES 256 CBC	2.221412771
Blowfish CBC	2.211554
DES CBC	1.84385782
Triple DES CBC	1.138862268
RC4	2.696817074

**Table 7.** Summary throughput results for secure communication (CIA).

**Device**	**Throughput**	**Throughput (Mb/s)**
X4	113 KB/s	0.882 Mb/s
X2	1.4 MB/s	11.2 Mb/s
Raspberry Pi, Command Line	2.962 MB/s	23.696 Mb/s
Raspberry Pi, Python Wrapper	1.5778 MB/s	12.6224 Mb/s
